# Site of Biopsy and Its Accuracy in Preoperative Diagnosis of Superficial Nonampullary Duodenal Epithelial Tumors: Retrospective Study

**DOI:** 10.3390/jcm14082579

**Published:** 2025-04-09

**Authors:** Yukihisa Fujinaga, Akira Mitoro, Hitoshi Mori, Satoshi Iwai, Takahiro Kubo, Misako Tanaka, Aritoshi Koizumi, Fumimasa Tomooka, Shohei Asada, Koh Kitagawa, Norihisa Nishimura, Shinya Sato, Kosuke Kaji, Tadashi Namisaki, Hitoshi Yoshiji

**Affiliations:** 1 Department of Gastroenterology, Nara Medical University, 840 Shijo-cho, Kashihara 634-8522, Nara, Japan; 2 Division of Endoscopy, Nara Medical University, 840 Shijo-cho, Kashihara 634-8522, Nara, Japan

**Keywords:** superficial nonampullary duodenal epithelial tumors, endoscopy, diagnosis, endoscopic mucosal resection

## Abstract

**Background/Objectives:** As endoscopy is increasingly being used to diagnose superficial nonampullary duodenal epithelial tumors (SNADETs), there is a growing need for their early detection and minimally invasive treatment. This study investigated the diagnostic accuracy of biopsy specimens for SNADETs. **Methods:** We conducted a retrospective analysis of clinicopathologic data from 98 patients with SNADETs who had undergone endoscopic resection. The presurgical diagnosis, based on biopsy specimens, was compared with the histological diagnosis of the excised specimens. **Results:** Herein, preoperative biopsies were performed on 98 SNADETs specimens from 91 patients. Of the 68 adenomas and 30 carcinomas, 22.4% adenomas were later found to be carcinomas. Carcinoma biopsy diagnosis sensitivity, specificity, and accuracy were 54.6%, 80.0%, and 71.4%, respectively. Biopsy accuracy for carcinoma differed significantly by location to the papilla of Vater (*p* = 0.0455). The preoperative biopsy diagnostics’ sensitivity, specificity, and accuracy for oral and anal carcinomas to Vater papilla were 69.2%, 92.0%, and 84.2% and 42.1%, 73.2%, and 63.3%, respectively. **Conclusions:** The diagnostic accuracy of biopsy for SNADETs was low; however, it was higher on the oral side than the anal side of the papilla of Vater. The biopsy of duodenal lesions should be performed after an endoscopic examination, considering their location and reducing the risks of fibrosis.

## 1. Introduction

Superficial nonpapillary duodenal epithelial tumors (SNADETs) are rare diseases, with a prevalence ranging from 0.3% to 4.6% [1,2,3]. However, advances in endoscopic technology have improved the diagnostic rate of SNADETs [4]. SNADETs consist of both superficial duodenal carcinomas and duodenal adenomas. Adenoma is considered a precancerous lesion because the adenoma–adenocarcinoma sequence has been observed in the duodenum [5]. As a result, SNADETs require early intervention such as endoscopic resection (ER) [6].

The duodenum is divided into bulbous, descending, horizontal and ascending portions. Embryologically, it is derived from the distal portion of the foregut and the proximal portion of at the midgut, with the transition between these anatomical structures located distal to the papilla of Vater. Anatomically, the duodenum is anatomically classified as part of the small intestine, and its mucosal surface is covered with small intestinal-type villous and crypt epithelium, similar to that of the jejunum and ileum. However, unlike the jejunum and ileum, the duodenum contains Brunner’s glands, which are endemic to this structure and located within the submucosa and mucosa. Brunner’s glands are densely distributed in the proximal duodenum but only sporadically presented in the distal portion. Brunner’s glands are composed of stable cells with properties similar to those of gastric mucous glands, yet they are capable of self-renewal and differentiation into gastric glandular epithelium when stimulated by erosion or ulceration. Gastric fundic gland-type cells (wall and principal cells) are also frequently observed within the duodenal mucosa and Brunner’s glands. Consequently, tumors of duodenal epithelial origin may have small intestinal-type traits, Brunner’s gland differentiation, or mixed traits.

ER techniques for SNADETs have advanced, and treatment options include endoscopic mucosal resection (EMR) and endoscopic submucosal dissection (ESD). In addition to conventional endoscopic mucosal resection (CEMR), EMR includes underwater EMR (UEMR), in which a duodenal lumen is filled with water or saline solution, and then a lesion is excised with a snare using a high-frequency current without local injection to submucosa [7]. ER techniques have advanced, with CEMR and underwater endoscopic mucosal resection (UEMR) showing promising results for tumors less than 20 mm in size, with few complications observed [8]. Complications of ER for duodenal tumors were noticed in 0.8% of cases and were represented by intraprocedural perforation and delayed bleeding in 2.6%, while delayed perforation was observed in 0.2% and delayed bleeding in 0.5% for CEMR. Overall, 0.2% of delayed perforation for EMR was detected along with 9.3% of intraprocedural perforation, 4.7% of delayed bleeding, and 2.3% of delayed perforation for ESD [7].

The presurgical diagnosis of SNADETs is critical in determining the course of treatment. However, studies comparing the presurgical diagnostic accuracy of biopsy with a histologic analysis of resected specimens found a low accuracy rate of 68–74% for the former [4,9,10]. Preoperative biopsy for SNADETs can rarely result in severe submucosal fibrosis, making CEMR difficult [10]. UEMR has made it possible to resect most SNADETs, even in the presence of fibrosis resulting from preoperative biopsies [11]. However, some cases required ESD to be performed due to these complications [11].

Biopsies can be used to accurately diagnose adenomas and carcinoid tumors [3]. Of the 221 lesions in which tissue biopsy in nonampullary duodenal elevated lesions were taken, 196 were non-neoplastic, and 25 were neoplastic, making the diagnosis of non-neoplastic lesions possible by biopsy [3].

As mentioned above, the disadvantage of biopsy is that the fibrosis present in a lesion may make treatment difficult, while the advantage is that it provides accurate diagnostics of whether it is non-neoplastic or neoplastic, and if non-neoplastic, it diminishes the risk of complications in ER.

To clarify the circumstances under which biopsy is acceptable in terms of the site, size, and macroscopic type of a tumor, we aimed to retrospectively evaluate the sensitivity, specificity, and accuracy of presurgical diagnostics by a biopsy of SNADETs.

## 2. Materials and Methods

### 2.1. Study Design

The current retrospective evaluation of the positive diagnostic rate of biopsy for duodenal tumors was conducted at the Department of Gastroenterology of Nara Medical University (Nara, Japan). This retrospective study was approved by the Institutional Review Board of Nara Medical University Hospital (approval no. 2049). This research was performed according to the Helsinki Declaration of the World Medical Association. Written informed consent was obtained from all patients before performing ER. Owing to the retrospective design of this study, an opt-out approach on the website was used instead of requiring written informed consent for inclusion in the sample.

### 2.2. Patients

Ninety-four patients with 102 lesions, who underwent CEMR or UEMR for SNA-DETs at the Department of Gastroenterology of Nara Medical University (Nara, Japan) between January 2016 and July 2023, were retrospectively assessed. Duodenal tumors measuring less than 20 mm were considered for ER. Four lesions in three patients were excluded because they were not biopsied prior to ER. Ninety-eight SNADETs from 91 patients who had presurgical biopsies were assessed for accuracy. The inclusion criteria were as follows: (1) age over 20 years old to 85 years old, (2) SNADETs with <20 mm, (3) a biopsy of SNADETs before ER, and (4) no previous surgical history of duodenal resection. The exclusion criteria were as follows: (1) patients who had opted out of the study because of the opt-out method used on the website, (2) patients with bleeding or perforation complications from biopsy or ER, (3) a serious underlying disease, and (4) previous distal gastrectomy.

### 2.3. Procedures

All ER procedures were performed by endoscopists certified by the Japan Gastroenterological Endoscopy Society and with extensive endoscopic treatment experience. ER was carried out using CEMR or UEMR. The presurgical diagnosis confirmed by biopsy specimen evaluation were compared to the histological results of the resected tissue. To examine the factors that led to the discrepancy between the pathology of the biopsy and the results of the post-treatment physiology, the groups with inconsistent biopsy and post-treatment pathology results (group for which results did not match) were compared to those with consistent results (group for which results matched).

### 2.4. Clinicopathological Evaluation

Clinical findings, such as age, gender, tumor size, location, macroscopic type, preoperative biopsy diagnoses, and the histologic analysis of resected specimens, were evaluated. Both specimens were examined by experienced pathologists. SNADETs were histologically classified into groups based on the histological grade represented by the adenoma and then further subclassified into low-grade dysplasia (LGD) or high-grade dysplasia (HGD) and superficial adenocarcinoma (SAC), which included carcinomas in situ and invasive carcinomas that invaded up to the submucosa.

### 2.5. Endpoints and Definitions

The primary endpoints were the sensitivity, specificity, and accuracy of presurgical diagnosis, confirmed by a histological analysis of biopsy specimens relative to the resected ones. The secondary endpoints were the examination of background factors in the groups in which the biopsy diagnosis matched the resection specimen diagnosis and those in which it did not and the sensitivity, specificity, and accuracy of presurgical diagnosis by duodenal site.

Collected data included the patients’ age, sex, lesion characteristics (location, size, and macroscopic type), number of performed CEMRs or UEMRs, and pathological diagnosis of biopsy specimens or resected specimens. A pathologic evaluation of the resected specimen, rather than that of preoperative biopsy, was considered to confirm the final diagnosis.

The Paris classification was used to differentiate macroscopic types. Moreover, 0-I is the elevated type, in which the lesion is elevated or raised above the surrounding mucosa. Additionally, 0-IIa is the superficial elevated type, in which a lesion is slightly elevated and still relatively flat but shows mild protrusion. Next, 0-IIc is the depressed type, in which a lesion is slightly depressed, still relatively flat, but has mild depression. Finally, 0-IIa+IIc is a combined elevated and depressed type, in which a lesion exhibits both characteristics.

### 2.6. Statistical Analysis

The descriptive statistics for all variables were shown as mean ± standard deviation or median (range) based on the distribution type. Quantitative data were analyzed using the Mann–Whitney U-test. Fisher exactor chi-square tests were used to analyze categorical data. All statistical analyses were performed using EZR (Version 1.68, Saitama Medical Center, Jichi Medical University, Saitama, Japan), a graphical user interface for R (The R Foundation for Statistical Computing, Vienna, Austria), which is a modified version of R with a broader command set for biostatistical analysis. Statistical significance was determined at *p* < 0.05.

## 3. Results

### 3.1. Characteristics of the Patients and the Lesions

In this study, ER was performed on 98 patients who had 102 SNADETs. Of those lesions, 98 SNADETs in 91 patients were biopsied before ER (19 cases involving the treatment with CEMR and 79 where UEMR had been conducted). There were no complications from biopsy or ER, such as bleeding or perforation. The clinicopathological characteristics of patients and lesions are shown in Table 1. The average age of the patients was 67 ± 10.4 years. There were 21 cases of lesions located in the first part of the duodenum, 70 in the second, and 7 in the third. The average size of all tumors was 9.8 ± 5.8 mm. Presurgical diagnosis using biopsy specimens revealed 68 adenomas and 30 carcinomas, while histological analysis after resection revealed 66 and 32, respectively.

### 3.2. Sensitivity, Specificity, and Accuracy of Presurgical Diagnosis by Biopsy

Both types of diagnosis corresponded to 17 cancerous lesions and 53 adenomas. However, of the 68 lesions diagnosed through biopsy sampling as adenomas, 15 (22.1%) were later confirmed as carcinomas (Table 2). The sensitivity, specificity, and accuracy of the presurgical diagnoses of carcinomas confirmed by biopsy were 53.1% (95% confidence interval [CI], 36.4–71.9), 80.3% (95% CI, 68.2–88.9), and 71.4% (95% CI, 61.4–80.1), respectively (Table 2).

### 3.3. Sensitivity, Specificity, and Accuracy of Presurgical Diagnosis by Biopsy Site

A comparison of the biopsy and post-treatment pathological analysis results between the non-matching and matching groups revealed significant differences on the oral and anal sides of the papilla of Vater (*p* = 0.0455) (Table 3). There were no significant differences in site (the first, second, and third part), tumor size, or tumor morphology between the two groups (Table 3). Table 4 shows the results of the biopsy and the excised specimen from the duodenum on the oral side of the papilla of Vater, and Table 5 shows the results of the biopsy and the excised specimen from the duodenum on the anal side of the papilla of Vater. The sensitivity, specificity, and accuracy of the biopsy-confirmed presurgical diagnosis of carcinomas on the oral and anal sides of the papilla of Vater were 69.2% (95% CI, 38.6–90.9), 92.0% (95% CI, 74.0–99.0), and 84.2% (95% CI, 68.7–94.0) and 42.1% (95% CI, 20.3–66.5), 73.2% (95% CI, 57.1–85.8), and 63.3% (95% CI, 49.9–75.4), respectively (Table 4, Table 5 and Table 6).

## 4. Discussion

In our study, the lesions diagnosed as neoplastic on biopsy were also diagnosed as this type requiring treatment in the resected specimens. The accuracy of biopsy-based diagnostics of SNADETs was 71.4%, indicating that its diagnostic ability is inadequate. Kakushima et al. investigated the accuracy of presurgical biopsy sampling in differentiating adenoma from adenocarcinoma, finding 74% accuracy, 72% sensitivity, and 80% specificity [9]. Kinoshita et al. and Goda et al. assessed the accuracy of presurgical biopsy in differentiating high-grade adenoma or adenocarcinoma from low-grade adenoma, with 72% and 68% accuracy, 38% and 58% sensitivity, and 93% and 93% specificity, respectively [4,10]. Thus, the accuracy of biopsy-based diagnostics of SNADETs was comparable to that of our study. The issue with this method is that a presurgical biopsy of SNADETs can result in severe submucosal fibrosis, making ER difficult in rare cases [10].

As a result, diagnostic methods based on image-enhanced or magnifying endoscopy are being studied. In a study on endoscopic diagnostics that used both the white light imaging (WLI) and image-enhanced magnifying endoscopy of SNADETs, Kakushima et al. and Ishii et al. evaluated the accuracy of presurgical biopsy for distinguishing high-grade adenoma or adenocarcinoma from low-grade adenoma. They found that the accuracy was 86% and 92%, the sensitivity was 63% and 95%, and the specificity was 96% and 93%, respectively [12,13]. Nakayama et al. indicated an accuracy of 65.1%, sensitivity of 90.5%, and specificity of 52.4% in distinguishing between high-grade adenoma or adenocarcinoma from low-grade adenoma by both WLI and image-enhanced magnifying endoscopy [14]. As previously mentioned, compared to biopsies, the accuracy of endoscopy has been demonstrated to be either higher or lower.

Adenomas, adenocarcinomas, and neuroendocrine tumors are examples of duodenal epithelial tumors, while duodenitis and non-neoplastic lesions such as ectopic gastric mucosa and Brunner’s gland hyperplasia are considered differential diagnoses. Sometimes, a biopsy may be useful in distinguishing between non- and neoplastic diseases [15]. ER for SNADETs is associated with complications, such as perforation and posterior hemorrhage, though at a low rate, and the resection of non-neoplastic lesions is discouraged. Kiguchi et al. demonstrated the utility of UEMR as a measure of non-lifting signs caused by biopsy scars [8,11].

When the biopsy and post-treatment pathological results were compared between the matched and unmatched groups, there were significant differences on the oral and anal sides of the papilla of Vater (*p* = 0.0455) (Table 3). The sensitivity, specificity, and accuracy of presurgical diagnostics by biopsy for carcinoma on the oral and anal sides from the papilla of Vater were 69.2% (95% CI, 38.6–90.9), 92.0% (95% CI, 74.0–99.0), and 84.2% (95% CI, 68.7–94.0) and 42.1% (95% CI, 20.3–66.5), 73.2% (95% CI, 57.1–85.8), and 63.3% (95% CI, 49.9–75.4), respectively (Table 4 and Table 5). Biopsies were performed with greater sensitivity, specificity, and accuracy on the oral side of the papilla of Vater than on the anal side of the anatomic structure. However, this indicates that there are approximately 30% false negative cases in the diagnostics of these lesions as well, and this should be kept in mind.

Embryologically, the foregut forms the esophagus, stomach, and proximal duodenum, while the midgut forms the distal duodenum and the proximal two-thirds of the transverse colon. The duodenum is embryologically made up of the distal portion of the foregut and the proximal portion of the midgut, with the transition located distal to the opening of the common bile duct. Intestinal-type tumors are distributed almost uniformly throughout the duodenum because of its small intestinal-type epithelial background. Gastric-type tumors are mostly present on the oral side of the papilla of Vater and less frequently on the anal side. Many pathologists diagnose intestinal-type adenomas and carcinomas using colorectal tumor criteria, whereas gastric-type adenomas and carcinomas are diagnosed using gastric tumor criteria. Although the cellular differentiation (trait expression) of nonpapillary duodenal tumors was not examined in this study, the differences in trait expression may have affected the accuracy of the biopsy of SNADETs.

**Figure 1:**
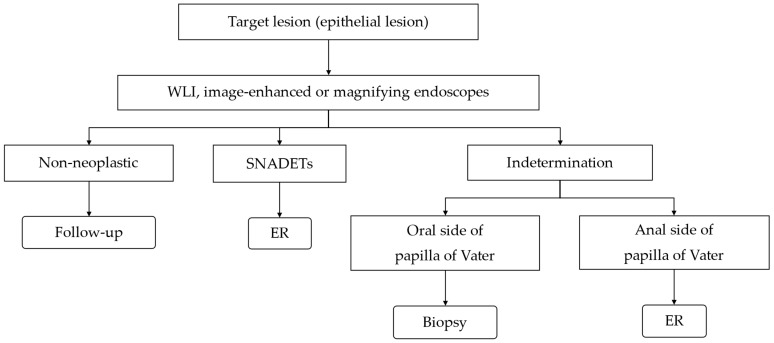
Flowchart of diagnosis and treatment of duodenal epithelial lesions.

Based on the above, we perform ER without biopsy if a lesion is considered neoplastic on endoscopic observation and consider biopsy if it is difficult to determine its nature. Figure 1 shows our flowchart of diagnosis and treatment for duodenal epithelial lesions.

Our study has several limitations. First, it was a single-center retrospective study with a relatively small number of patients. Second, background factors for the groups in which the biopsy and post-treatment pathological analysis results did not match and for those in which they did were not adjusted. Third, the sensitivity, specificity, and accuracy of neoplastic or non-neoplastic lesions detected by biopsy was not examined. Finally, the volume of biopsy specimens, which may have affected the pathological diagnosis of a biopsy, was not considered.

## 5. Conclusions

Although the accuracy of the biopsy of SNADETs was relatively low, the sensitivity, specificity, and accuracy of that by site were higher on the oral side rather than on the anal side of Vater papillae. As biopsy-induced fibrosis can affect the outcomes of treatment, a biopsy of duodenal lesions should be performed after considering the location when it is difficult to confirm whether it is neoplastic by endoscopic observation using image-enhanced or magnifying endoscopes.

## Figures and Tables

**Table 1:** Clinicopathological characteristics of 91 patients and 98 lesions.

	*n* = 91 (98 Lesions)
Gender, male or female (*n* = 91)	65:26
Age, yr (*n* = 91)	67 ± 10.4
Location (98 lesions)	
First part	21
Second part	70
Third part	7
Macroscopic type (98 lesions)	
0-I	15
0-IIa	58
0-IIc	12
0-IIa+IIc	13
Treatment (98 lesions)	
CEMR	19
UEMR	79
Presurgical diagnosis based on biopsy specimen (98 lesions)	
Adenoma	68
Carcinoma	30
Histologic diagnosis of resected specimens (98 lesions)	
Adenoma	66
Carcinoma	32

Data are presented as mean ± SD or number (%).

**Table 2:** Comparison of presurgical diagnoses made by analysis of biopsy specimens and histology of resected specimens.

		Histological Evaluation of Resected Specimens		
		Carcinoma	Adenoma	
Presurgical diagnosis confirmed by the analysis of biopsy specimens	Carcinoma	17	13	30
	Adenoma	15	53	68
		32	66	98

**Table 3:** Clinical characteristics of groups with and without concordant biopsy and post-treatment pathology results.

	Group for Which Results Did Not Match (28 lesions)	Group for Which Results Matched (70 lesions)	*p*-Value
Location 1			0.4776
First part	3 (15.0)	17 (85.0)	
Second part	22 (31.0)	49 (69.0)	
Third part	3 (42.9)	4 (57.1)	
Location 2			0.0455
Oral side of papilla of Vater	6 (16.0)	32 (84.0)	
Anal side of papilla of Vater	22 (37.7)	38 (63.3)	
Tumor size, mm: Mean ± SD	9.1 ± 6.3	10.0 ± 5.6	0.4957
Macroscopic type			0.3025
0-I	2 (14.3)	12 (85.7)	
0-IIa	20 (40.8)	29 (59.2)	
0-IIc	4 (33.3)	8 (66.7)	
0-IIa+IIc	2 (15.4)	11 (84.6)	

Data are presented as mean ± SD or number (%).

**Table 4:** Comparison of presurgical diagnosis based on biopsy specimens and histologic analysis of resected specimens on oral side of papilla of Vater.

Oral side of Papilla of Vater		Histological Evaluation of Resected Specimens		
		Carcinoma	Adenoma	
Presurgical diagnosis con-firmed by the analysis of biopsy specimens	Carcinoma	9	2	11
	Adenoma	4	23	27
		13	25	38

**Table 5:** Comparison of presurgical diagnosis made by analysis of biopsy specimens and histology of resected specimens on anal side of papilla of Vater.

Anal side of Papilla of Vater		Histological Evaluation of Resected Specimens		
		Carcinoma	Adenoma	
Presurgical diagnosis con-firmed by the analysis of biopsy specimens	Carcinoma	8	11	19
	Adenoma	11	30	41
		19	41	60

**Table 6:** The sensitivity, specificity, and accuracy of the biopsy-confirmed presurgical diagnosis of carcinoma on the oral and anal sides of the papilla of Vater.

	Oral Side of Papilla of Vater	Anal Side of Papilla of Vater
Sensitivity (%)	69.2	42.1
Specificity (%)	92.0	73.2
Accuracy (%)	84.2	63.3

## Data Availability

Data are contained within the article.

## Abbreviations

The following abbreviations are used in this manuscript:

SNADETs	Superficial nonampullary duodenal epithelial tumors
ER	Endoscopic resection
CEMR	Conventional endoscopic mucosal resection
UEMR	Underwater endoscopic mucosal resection
LGD	Low-grade dysplasia
HGD	High-grade dysplasia
SAC	Superficial adenocarcinoma
